# Effects of High-Intensity Interval Training on Body Composition and Cardiometabolic Health in Physically Inactive Individuals: A Systematic Review and Meta-Analysis of Randomized Controlled Trials

**DOI:** 10.3390/metabo16070514

**Published:** 2026-07-22

**Authors:** Cunyu Lan, Beibei Lei, Juerui Li, Wenjie Huang, Kun Yang, Bo Gou

**Affiliations:** 1 Department of Sport Rehabilitation, School of Graduate Studies, Xi’an Physical Education University, Xi’an 710068, China; 18078606451@163.com (C.L.);; 2 Rehabilitation Medicine College, Henan University of Chinese Medicine, Zhengzhou 450046, China; 3 Tianjin Key Laboratory of Exercise Physiology and Sports Medicine, Institute of Sport, Exercise & Health, Tianjin University of Sport, Tianjin 301617, China; 4 Key Laboratory of Sports Technology Analysis and Skill Assessment (Xi’an Physical Education University), General Administration of Sport, Xi’an 710068, China

**Keywords:** sedentary behavior, cardiorespiratory fitness, blood pressure, lipid profile, exercise, public health

## Abstract

**Highlights:**

**What are the main findings?**

- HIIT was associated with improvements in selected body composition and cardiometabolic outcomes, with a relatively consistent direction of effect for VO_2_max.
- Certainty of evidence was low or very low, and some findings were not robust, warranting confirmation in larger future studies.

**What are the implications of the main findings?**

- HIIT may offer a time-efficient exercise option for physically inactive individuals, although its equivalence or superiority to MICT remains uncertain.
- Current evidence is insufficient to support recommendations for an optimal HIIT intervention protocol.

**Abstract:**

**Background/Objectives:** Increasing physical activity is important for reducing cardiometabolic risk. However, evidence on the effects of high-intensity interval training (HIIT) in physically inactive individuals, including those with extremely sedentary behavior, remains inconsistent. We evaluated the effects of HIIT on body composition and cardiometabolic outcomes compared with a non-exercise control (CON) and moderate-intensity continuous training (MICT). **Methods:** PubMed, Web of Science, Embase, the Cochrane Library, Ovid-hosted resources, and ClinicalTrials.gov were searched through 8 July 2026. The protocol was registered (PROSPERO CRD420251230848). Random-effects models were used. Risk of bias, evidence certainty, and robustness were assessed using the Cochrane Risk of Bias 2 (RoB 2) tool, Grading of Recommendations Assessment, Development and Evaluation (GRADE), and leave-one-out analyses, respectively. **Results:** Twenty-four randomized trials involving 877 participants were analyzed. A conservative study-level summary classified all studies as having at least some concerns, including five at high risk of bias. Compared with CON, HIIT reduced body weight (weighted mean difference [WMD] = −1.60 kg), systolic blood pressure (WMD = −2.85 mmHg), diastolic blood pressure (WMD = −6.57 mmHg), low-density lipoprotein cholesterol (WMD = −0.29 mmol/L), and total cholesterol (WMD = −0.42 mmol/L), and increased maximal oxygen uptake (VO_2_max; WMD = 7.72 mL/kg/min). Compared with MICT, most outcomes showed no statistically significant differences, but HIIT produced a greater VO_2_max increase (WMD = 3.72 mL/kg/min). Several findings were sensitive to individual-study removal, and certainty of evidence was low or very low. No serious adverse events were reported in 11 studies; attendance or adherence was generally high in 15 studies, despite incomplete reporting and supervised interventions. **Conclusions:** HIIT may improve selected body composition and cardiometabolic outcomes, with VO_2_max showing a relatively consistent direction of effect. However, the certainty of evidence for most outcomes was low or very low; therefore, these findings should be interpreted with caution. Current evidence is insufficient to establish equivalence to MICT or identify an optimal HIIT protocol. Larger, longer-term, high-quality trials using objective measures of sedentary behavior are needed, particularly in extremely sedentary populations.

## 1. Introduction

Physical inactivity and sedentary behavior are related but distinct constructs. Physical inactivity is defined as performing an insufficient volume of moderate-to-vigorous physical activity to meet current guidelines, whereas sedentary behavior refers to waking activities performed in a sitting, reclining, or lying posture with very low energy expenditure (≤1.5 metabolic equivalents) [1,2]. Physical inactivity is associated with higher mortality from cardiovascular disease and cancer and has become a global public health problem across all age groups [3]. In 2022, the age-standardized prevalence of physical inactivity among adults worldwide reached 31.3%, affecting approximately 1.8 billion people, with a higher prevalence in women than in men (33.8% vs. 28.7%) [4]. In 2016, more than 80% of school-going adolescents aged 11–17 years worldwide did not meet the recommendation of an average of at least 60 min of moderate-to-vigorous physical activity per day, and girls were less active than boys in most countries [5]. The burden is also substantial among middle-aged and older adults. Across regions, the prevalence of physical inactivity is generally highest in the oldest age groups and increases markedly after 60 years of age [4]. These findings indicate that physical inactivity occurs across the life course, affecting children, adolescents, adults, and older populations. In developed countries, adults spend an average of approximately 8.2 h per day in sedentary pursuits. Sedentary behavior and physical inactivity frequently coexist, particularly among working adults and students [2,4]. Based on the framework proposed by Rey-Brandariz et al. (2023) [6], individuals who exhibit both sedentary behavior and physical inactivity are conceptualized as “extremely sedentary.” In the present review, this term refers to individuals for whom both behaviors were documented. Because sedentary exposure was not consistently quantified across the primary studies, this population was examined as an exploratory subgroup rather than treated as a uniformly defined population. Individuals with both behaviors may face elevated risks of cardiometabolic disease and all-cause mortality. Accordingly, jointly assessing sedentary behavior and physical inactivity may better characterize health risk than evaluating either behavior alone, thereby facilitating more targeted interventions [1,2,3]. Beyond their combined effects, physical inactivity, particularly when combined with prolonged sedentary behavior, is an important modifiable risk factor for cardiometabolic disease. Over time, insufficient physical activity may reduce energy expenditure, promote fat accumulation, and contribute to elevated blood pressure, impaired glucose regulation, and adverse lipid profiles, thereby increasing cardiometabolic risk [7]. Measures of body composition, blood pressure, glucose and lipid metabolism, and cardiorespiratory fitness can reflect these changes before major clinical outcomes, such as myocardial infarction or death, occur. For example, body mass index (BMI) and body fat percentage (BF%) reflect overall adiposity, whereas waist-to-hip ratio reflects central fat distribution. Blood pressure and blood glucose (BG) indicate hemodynamic load and glycemic status, whereas high-density lipoprotein cholesterol (HDL-C), low-density lipoprotein cholesterol (LDL-C), total cholesterol (TC), and triglycerides (TG) provide information on lipid metabolism and atherosclerotic risk [8,9]. Maximal oxygen uptake (VO_2_max) is a key measure of cardiorespiratory fitness. It reflects the integrated ability of the respiratory, circulatory, and skeletal muscle systems to take up, transport, and use oxygen and is closely associated with the risks of cardiovascular disease and all-cause mortality [10]. Therefore, these indicators are useful for early risk identification, evaluating intervention effects, and developing targeted public health strategies. Evidence suggests that sufficient physical activity can attenuate or even offset the adverse effects associated with prolonged sitting [4]. Nevertheless, physically inactive individuals commonly identify limited leisure time as a primary barrier to engaging in physical activity [6]. Identifying effective and time-efficient strategies to increase physical activity is therefore an urgent priority, particularly for individuals who have been physically inactive for prolonged periods and face time constraints.

Against this background, high-intensity interval training (HIIT), which consists of brief alternating bouts of vigorous exercise and active or passive recovery, has gained prominence as a time-efficient exercise modality [11]. Previous reviews suggest that HIIT may improve cardiorespiratory fitness in adults, including apparently healthy adults and adults with metabolic syndrome [12,13]. Potential mechanisms include central cardiovascular adaptations, skeletal muscle mitochondrial and capillary remodeling, and changes in metabolic and inflammatory signaling [14,15]. Repeated high-intensity bouts may also induce a marked acute metabolic disturbance and increase excess post-exercise oxygen consumption (EPOC), which reflects the elevated oxygen demand associated with recovery processes such as the restoration of ATP–phosphocreatine and oxygen stores, thermoregulation, and the return of cardiorespiratory and metabolic function toward resting levels. The magnitude and duration of EPOC vary according to exercise intensity, duration, total work, and recovery structure and may be greater after some HIIT protocols than after moderate-intensity continuous training (MICT). However, EPOC is an acute recovery response, and its contribution to long-term changes in body composition and cardiometabolic health remains uncertain [16]. HIIT may also demonstrate acceptable adherence and tolerability in physically inactive and sedentary populations and may be perceived as more enjoyable than MICT [17,18,19].

However, evidence in physically inactive populations remains fragmented across outcomes and population subgroups [20,21]. Evidence regarding safety also remains incomplete, because adverse events have not been consistently monitored or reported [22]. Several studies have examined the effects of HIIT on body composition and cardiometabolic risk factors in physically inactive individuals, but their findings remain inconsistent, potentially because of small sample sizes and heterogeneous protocols. For example, one study reported a reduction in BF% after HIIT, whereas another reported an increase, and both were restricted to specific age groups [23,24]. Similar discrepancies have been observed in previous evidence syntheses. A recent umbrella review identified discrepancies among earlier reviews and found only a small additional reduction in BF% compared with MICT, but no significant between-group differences in most other body composition outcomes [25]. Another meta-analysis in adults with overweight or obesity found no significant differences between HIIT and MICT in body weight (BW), BMI, waist circumference, BF%, or fat mass, although insulin sensitivity favored HIIT [26]. Many earlier meta-analyses focused primarily on clinical populations rather than physically inactive individuals as a distinct target population. Two earlier reviews that included sedentary participants had important limitations. One predominantly enrolled sedentary individuals with obesity alongside other clinical populations, lacked a non-exercise control group, and evaluated only body composition and cardiorespiratory fitness, without assessing lipid or glucose outcomes [27]. The other included metabolic outcomes but focused on individuals at risk of diabetes, with relatively few studies involving physically inactive participants [20]. More recent reviews focusing specifically on sedentary populations also left important gaps. A 2025 meta-analysis included 14 studies, but its quantitative synthesis did not assess body composition, blood glucose, or lipid outcomes. It also combined relatively healthy sedentary participants with clinical or high-risk populations, including individuals with diabetes, elevated cardiovascular risk, and cancer survivors. The small number of studies and incomplete reporting of the original data further limited detailed subgroup analysis [21]. Another meta-analysis systematically evaluated HIIT in apparently healthy sedentary adults aged 18–64 years. However, the exclusion of adolescents limited the ability to determine whether its findings could be generalized to younger physically inactive populations. The review also did not comprehensively synthesize several clinically relevant outcomes, including BMI, body fat percentage, and the full lipid profile. Furthermore, because none of the included studies reported adverse-event data and it was unclear whether adverse events had been actively monitored, the safety of HIIT could not be adequately evaluated [22]. These two meta-analyses of sedentary populations also yielded inconsistent findings for blood pressure outcomes, which may partly reflect methodological differences between the reviews. Overall, the effects of HIIT may vary according to the population, comparator, and outcome assessed. Previous evidence remains fragmented across populations, comparators, and outcomes, and extremely sedentary individuals have not been consistently examined as a distinct subgroup.

Therefore, this systematic review and meta-analysis aimed to evaluate the effects of HIIT on body composition, blood pressure, glucose and lipid metabolism, and cardiorespiratory fitness in physically inactive individuals. We also compared HIIT with MICT, synthesized evidence on safety and adherence, and conducted exploratory subgroup analyses to examine whether the observed effects appeared to vary according to selected participant and intervention characteristics, including extremely sedentary status. We hypothesized that HIIT would improve body composition and cardiometabolic health and produce effects comparable to or greater than those achieved with MICT, although the magnitude of these effects might vary according to participant characteristics and intervention protocols. If supported, these findings could provide physically inactive individuals with an additional time-efficient exercise option.

## 2. Materials and Methods

### 2.1. Protocol Registration and Amendments

This systematic review and meta-analysis followed the PRISMA 2020 guidelines and was prospectively registered in PROSPERO (CRD420251230848). Several deviations from the originally registered protocol arose during the conduct of the review and are reported below. A major amendment documenting these changes was submitted to PROSPERO on 15 July 2026. First, the original eligibility criterion of 18–75 years was expanded to 10–75 years to enable a broader evaluation of physically inactive individuals across age groups and to include adolescents. This change reflected an expansion of the review scope and was not based on observed outcome effects. Second, the PROSPERO record prespecified VO_2_max and BMI as primary outcomes and BW and adverse events as secondary outcomes. BF%, waist-to-hip ratio (WHR), systolic blood pressure (SBP), diastolic blood pressure (DBP), BG, TC, TG, HDL-C, LDL-C, and adherence were not explicitly listed in the original registration record. After study selection and at the beginning of data extraction, these additional outcomes were identified as relevant to the review question and the prespecified domains of body composition and cardiometabolic health. They were therefore added as post hoc additional secondary outcomes and extracted using the same standardized procedures. This decision was made before quantitative synthesis and was not based on the direction or statistical significance of the results. Adverse events and adherence were summarized descriptively. An outcome was included in the quantitative synthesis only when data were available from at least three studies. Subgroup analyses by age, baseline weight status, physical activity level, total session duration, training frequency, and intervention duration were not prespecified in the original PROSPERO record. These subgroup analyses were defined after study selection based on differences in participant and intervention characteristics, rather than on observed outcome effects, and were therefore considered exploratory. Third, the protocol-specified approach to selecting the meta-analytic model based on statistical heterogeneity was revised. Random-effects models were used for all main meta-analyses because clinical and methodological heterogeneity was anticipated across studies, including differences in HIIT protocols, populations, comparators, and intervention durations. Fourth, all database searches were updated through 8 July 2026. ClinicalTrials.gov was added as a supplementary source to identify ongoing or completed trials and any related eligible publications. The ClinicalTrials.gov search was conducted on 8 July 2026, and the full search strategy is provided in Supplementary File S1. This search identified 133 trial records but no additional eligible peer-reviewed full-text publications.

### 2.2. Search Strategy

PubMed, Web of Science, Embase, the Cochrane Library, resources available through the Ovid platform, and ClinicalTrials.gov were searched from inception through 8 July 2026. The search combined subject headings and keywords, including “high-intensity interval training,” “sprint interval training,” “physical inactivity,” and “sedentary behavior,” among others. The detailed search strategy is provided in Supplementary File S1. No language restrictions were applied. Only peer-reviewed full-text articles were eligible. Conference abstracts and proceedings were excluded because they did not provide sufficient information for eligibility assessment, risk-of-bias assessment, and quantitative synthesis. Review articles were not eligible for inclusion, but their reference lists were screened to identify potentially relevant studies. ClinicalTrials.gov records were used to identify ongoing or completed trials and any linked eligible publications; registry records without an eligible full-text publication were not treated as eligible studies or included in the quantitative synthesis.

### 2.3. Eligibility Criteria

Eligibility criteria were prespecified for study selection. Studies were included if they: (1) were randomized controlled trials; (2) enrolled participants aged 10–75 years because physical inactivity represents a substantial health concern across different stages of the lifespan, and the review aimed to comprehensively evaluate the effects of HIIT in physically inactive individuals across these stages; (3) enrolled physically inactive participants, with physical inactivity defined as activity levels below age-appropriate WHO or national recommendations (<60 min/day of moderate-to-vigorous physical activity for adolescents; <150 min/week of moderate-intensity or <75 min/week of vigorous-intensity activity for adults), an International Physical Activity Questionnaire (IPAQ) score of <600 MET-min/week, or no regular exercise during the preceding 6–12 months; (4) evaluated HIIT comprising repeated high-intensity bouts interspersed with active or passive recovery, with intensity prescribed at ≥85% of maximum heart rate (HRmax), ≥80% of VO_2_max, or an equivalent prespecified high-intensity criterion; (5) compared HIIT with no exercise intervention or MICT; and (6) reported at least one extractable outcome related to body composition, blood pressure, glucose or lipid metabolism, or cardiorespiratory fitness. Functional circuit-based HIIT was eligible if it retained this intermittent high-intensity structure. Studies were excluded if they were non-human studies, conference abstracts or proceedings, reviews, protocols, or publications without ex-tractable data. Studies combining HIIT with another independent intervention were also excluded when the effect of HIIT could not be isolated. Functional or body-weight exercises incorporated within the HIIT protocol were not considered separate interventions.

### 2.4. Study Selection

After duplicate removal using EndNote X9, two reviewers independently screened the titles and abstracts, followed by full-text assessment. The reviewers were not blinded to one another’s decisions, and any disagreements were resolved through consultation with a third reviewer. No additional software was used for study screening. When full texts were unavailable, attempts were made to contact the corresponding authors by email. In addition, the reference lists of included studies were manually screened to identify potentially eligible studies that might have been missed in the initial database search.

### 2.5. Data Extraction

Two investigators independently extracted data using a standardized data extraction form, and all discrepancies were resolved through consultation with a third investigator. Extracted study characteristics included the author and year of publication, participant characteristics, and intervention characteristics, including exercise modality, HIIT type, intervention duration, training frequency, and session duration. Baseline and post-intervention means and standard deviations were extracted for body composition, blood pressure, glucose and lipid metabolism, and cardiorespiratory fitness outcomes. Adverse events and attendance rates were also extracted. The prespecified primary outcomes were VO_2_max and BMI, whereas BW and adverse events were prespecified as secondary outcomes. All other eligible outcomes were treated as post hoc additional secondary outcomes, as described in Section 2.1.

### 2.6. Risk of Bias Assessment

Two reviewers independently assessed the risk of bias using the Cochrane RoB 2 tool for individually randomized parallel-group trials. The target of the assessment was the effect of assignment to the intervention. Separate risk-of-bias judgments were made for each available post-intervention result, including BW, BMI, BF%, WHR, SBP, DBP, TG, TC, HDL-C, LDL-C, BG, and VO_2_max. For each assessed result, the overall risk of bias was judged as low risk, some concerns, or high risk. Any disagreements were resolved through discussion between the two reviewers, with a third reviewer consulted when necessary. Detailed outcome-specific judgments are provided in the Supplementary Materials Table S3. For concise presentation in the main text, an additional study-level summary was generated using the most conservative overall judgment across the assessed outcomes within each study. This study-level summary was used for presentation only and did not replace the outcome-specific RoB 2 assessments.

### 2.7. Statistical Analysis

Review Manager 5.4.1 was used to perform data synthesis, subgroup analyses, and sensitivity analyses. For continuous outcomes, weighted mean differences (WMDs) with 95% confidence intervals (CIs) were calculated. WMDs provide direct estimates of intervention effects on the original measurement scales and facilitate clinical interpretation. Change-from-baseline data were used in the pooled analyses. When not directly reported, mean changes were calculated as post-intervention means minus baseline means using Equation (1), and the corresponding change-score SDs were estimated using Equation (2), with r assumed to be 0.5, a commonly used value [28]. Blood glucose and lipid outcomes were reported using different units across the included studies. For the pooled analyses, all values reported in conventional units (mg/dL) were converted to international units (mmol/L). Specifically, BG was converted from mg/dL to mmol/L by dividing by 18; TC, HDL-C, and LDL-C by 38.67; and TG by 88.57. All pooled analyses of these outcomes were performed using the converted mmol/L values. Statistical heterogeneity was assessed using the I^2^ statistic. Given the anticipated clinical heterogeneity arising from differences in participant characteristics, baseline physical activity levels, and HIIT intervention protocols across the included studies, random-effects models were used for all meta-analyses. Meta-analyses were conducted separately for two comparisons: HIIT versus non-exercise control (CON) and HIIT versus MICT. For outcomes reported by at least 10 studies, potential publication bias was assessed by visual inspection of funnel plots generated in Stata 15.0. The robustness of the pooled results was examined using leave-one-out sensitivity analyses.

Based on differences in intervention protocols and participant characteristics among the included studies, exploratory subgroup analyses were conducted to investigate potential sources of heterogeneity. Subgroups were defined according to intervention duration (<12 weeks vs. 12 weeks), intervention frequency (low: 2–3 sessions/week; high: 4–5 sessions/week), total session duration (≤30 min/session vs. >30 min/session), physical activity status (insufficient physical activity alone vs. extremely sedentary), age group [adolescents (<20 years), young adults (20–39 years), and middle-aged to older adults (≥40 years)], and baseline weight status (overweight/obesity vs. normal weight). Age groups were assigned according to the reported mean age or the midpoint of the reported age range, and weight status was determined using baseline BMI. Total session duration was defined as the time from the start of the warm-up to the end of the cool-down, including both the interval-training and recovery periods. This measure reflected participants’ total training exposure and time commitment for a complete HIIT session, rather than the accumulated duration of the high-intensity bouts alone. Participants in the extremely sedentary subgroup were required to meet the criteria for insufficient physical activity and to exhibit sedentary behavior. Sedentary behavior was determined from information reported in the original studies, including explicit descriptions of participants as sedentary, questionnaire-based assessments, or reported sedentary time. Because the original studies used heterogeneous definitions and often did not report a uniform sedentary-time threshold, the extremely sedentary classification was considered exploratory. All subgroup findings were interpreted as exploratory, and subgroup estimates based on fewer than three studies were interpreted with particular caution.

(1) $${\bar{X}}_{change}={\bar{X}}_{final}-{\bar{X}}_{baseline}$$

(2) $$SD_{\mathrm{change}}=\sqrt{SD_{\mathrm{baseline}}^{2}+\;SD_{\mathrm{final}}^{2}-\;2r\cdot SD_{\mathrm{baseline}}\;\cdot \;SD_{\mathrm{final}}}$$

In these equations, ${\bar{X}}_{change}$ represents the mean change from baseline, ${\bar{X}}_{final}$ represents the post-intervention mean, and ${\bar{X}}_{baseline}$ represents the baseline mean. *SD_change_*, *SD_baseline_*, and *SD_final_* represent the standard deviations of the change score, baseline measurements, and post-intervention measurements, respectively. The term *r* represents the assumed correlation coefficient between baseline and post-intervention measurements.

### 2.8. Certainty of Evidence Assessment

The certainty of evidence for each outcome was assessed using the Grading of Recommendations Assessment, Development and Evaluation (GRADE) framework. Two independent reviewers assessed each outcome across the five GRADE domains: risk of bias, inconsistency, imprecision, indirectness, and publication bias. The overall certainty of evidence for each outcome was rated as high, moderate, low, or very low. Any disagreements between the reviewers were resolved through consensus, with a third researcher consulted when necessary. Specific reasons for each downgrading decision are provided in the summary of findings tables.

## 3. Results

### 3.1. Study Selection

**Figure 1:**
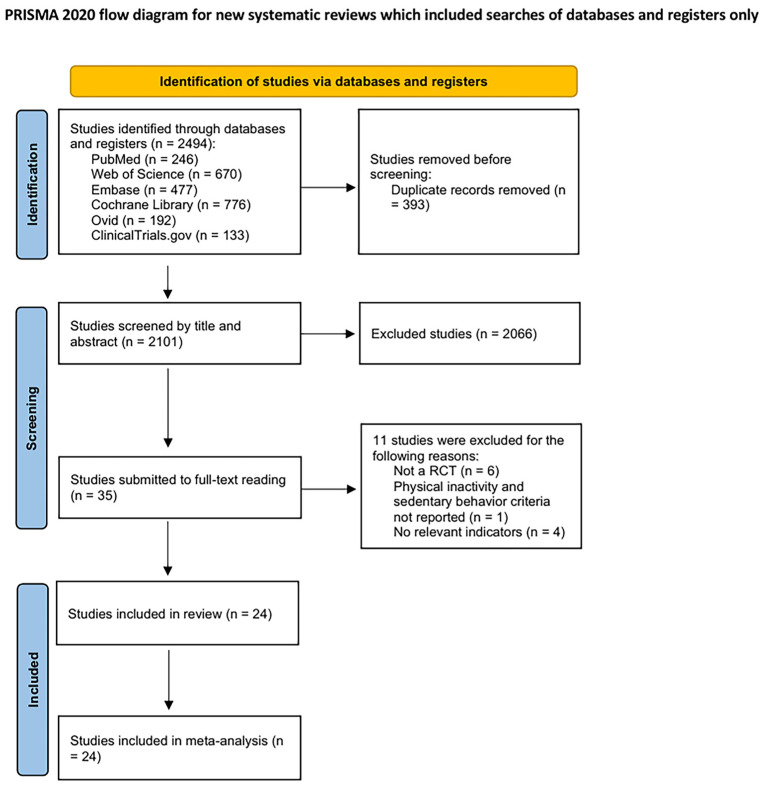
PRISMA flow diagram of the study selection process.

A total of 2494 records were identified from databases and registers. After the removal of 393 duplicate records, 2101 records remained for title and abstract screening. After screening, 2066 records were excluded, and 35 full-text articles were assessed for eligibility. Finally, 24 studies [23,24,29,30,31,32,33,34,35,36,37,38,39,40,41,42,43,44,45,46,47,48,49,50] met the inclusion criteria and were included in the meta-analysis. The study selection process is shown in Figure 1.

### 3.2. Study Characteristics

Author information, publication years, and participant characteristics are summarized in Supplementary Table S1. A total of 877 physically inactive individuals were included. Among participants with reported sex, 522 were male and 288 were female; two studies did not report sex distribution [37,40]. The corresponding male-to-female ratio was approximately 1.81:1. Participant ages ranged from 16 to 67 years. Based on the reported BMI data, 397 participants were classified as overweight or obese and 439 were classified as normal weight. Only one study did not report BMI values [23]. Fifteen studies were classified, for exploratory purposes, as involving extremely sedentary populations based on the information reported in the original studies [24,30,32,34,35,39,40,41,42,43,44,45,46,47,49]. Nine trials focused on physical inactivity alone and included 402 participants [23,29,31,33,36,37,38,48,50]. The included studies were conducted in China (*n* = 10), the United States (*n* = 3), Australia (*n* = 2), Colombia (*n* = 2), Thailand (*n* = 1), Iran (*n* = 1), Spain (*n* = 1), the United Kingdom (*n* = 1), Japan (*n* = 1), Canada (*n* = 1), and Cyprus (*n* = 1). The outcomes assessed in the included studies comprised BW, BMI, SBP, DBP, BF%, VO_2_max, WHR, TG, BG, TC, HDL-C, and LDL-C. The outcomes reported by each study are detailed in Supplementary Table S1.

### 3.3. Intervention Characteristics

Intervention protocol characteristics are summarized in Supplementary Table S1. Intervention duration ranged from 2 to 12 weeks. Fourteen studies used cycling for HIIT [29,32,33,34,37,39,40,41,42,43,45,46,47,48], eight used running [24,30,31,35,37,38,44,49], one used a combination of agility ladder, resistance bands, dumbbells, and kettlebells [23], and one used swimming [50]. Regarding HIIT type, eight studies used sprint-based HIIT, seven used short-interval aerobic HIIT, seven used long-interval aerobic HIIT, one used circuit-based functional HIIT [23], and one used mixed-mode HIIT [44]. One study combined HIIT with placebo [24]. Only one study combined supervised and home-based training [23]; all other studies used supervised training. Eight studies compared HIIT with MICT, ten compared HIIT with CON, and six compared HIIT, MICT, and CON. The mean duration of a single HIIT session was approximately 30 min (range 2–60 min), compared with approximately 43 min for a single MICT session (range 30–65 min). Only two studies [33,42] prescribed MICT 5 sessions/week and HIIT 3 sessions/week; in the remaining studies, weekly session frequency was identical for MICT and HIIT. Nine studies used unequal exercise volumes, with lower total load or energy expenditure for HIIT than for MICT, whereas five studies matched exercise volume, with comparable total load or expenditure between HIIT and MICT. Detailed intervention protocols and design characteristics for studies comparing HIIT with MICT are provided in Supplementary Table S2.

### 3.4. Risk of Bias Assessment Results

**Figure 2:**
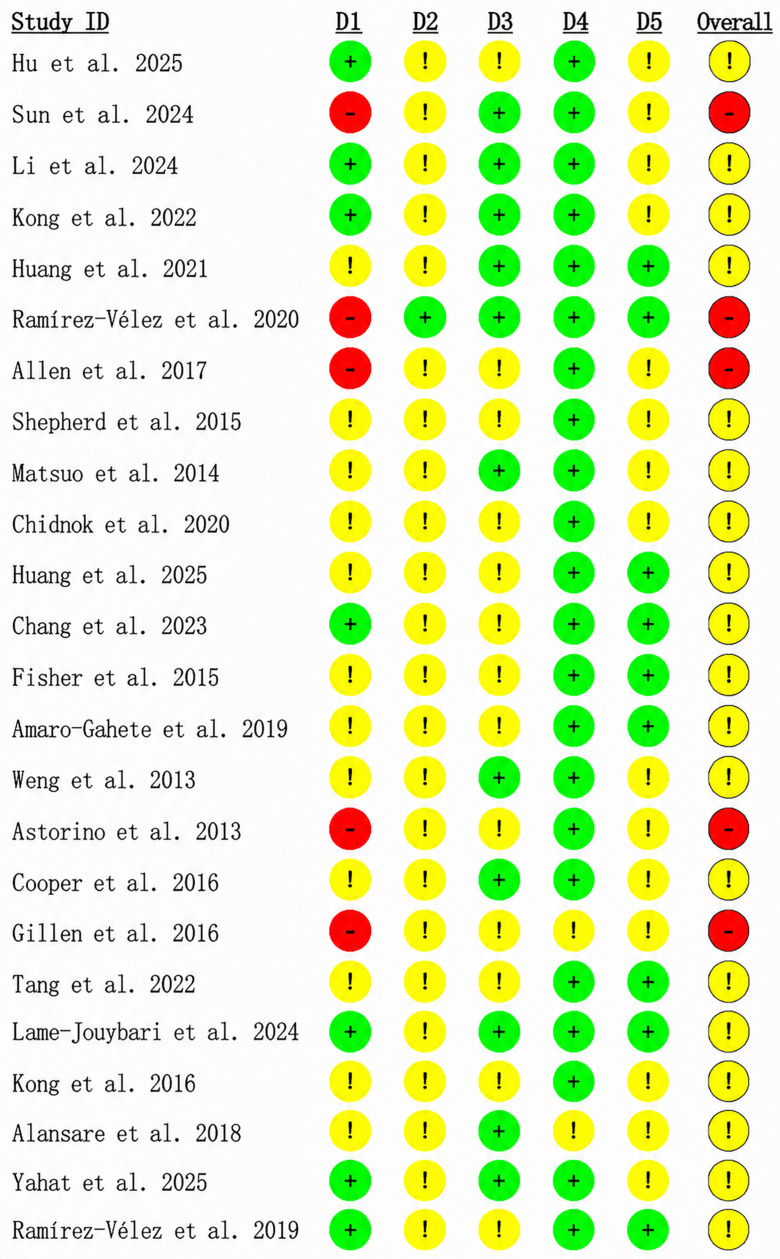
Conservative study-level summary of outcome-specific RoB 2 judgments. The studies presented in the figure correspond, from top to bottom, to references [23,24,29,30,31,32,33,34,35,36,37,38,39,40,41,42,43,44,45,46,47,48,49,50]. Green, yellow, and red indicate low risk of bias, some concerns, and high risk of bias, respectively. D1–D5 represent the five RoB 2 domains: bias arising from the randomization process, bias due to deviations from intended interventions, bias due to missing outcome data, bias in measurement of the outcome, and bias in selection of the reported result.

**Figure 3:**
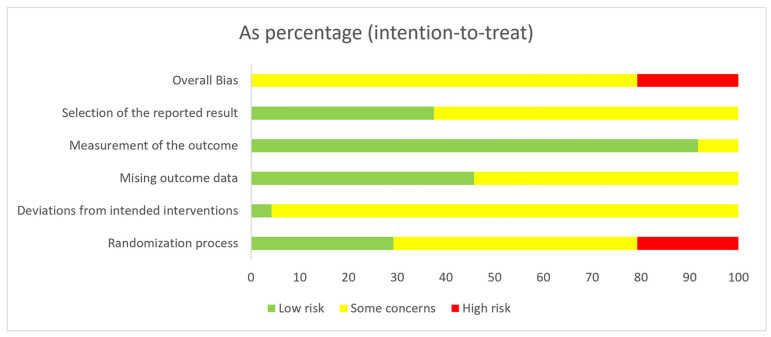
Distribution of conservative study-level risk-of-bias judgments across the five RoB 2 domains. Outcome-specific assessments were summarized at the study level using the more conservative judgment when judgments differed across outcomes. The proportions of the 24 studies judged as low risk, some concerns, or high risk of bias are presented for each domain. Green, yellow, and red indicate low risk of bias, some concerns, and high risk of bias, respectively.

Risk-of-bias assessments conducted using the Cochrane RoB 2 tool are presented in Figure 2. Separate outcome-specific risk-of-bias judgments were first made for all available outcomes within each study. Because the judgments were generally consistent across outcomes within the same study, an additional conservative study-level summary is presented for clarity. For each domain and the overall judgment, when outcomes within the same study differed, the higher risk judgment was used in the study-level display. In this conservative study-level summary, all 24 studies were categorized as having at least some concerns, including five categorized as being at high risk of bias [30,32,37,45,47]. The main sources of concern or high risk were deviations from intended interventions, missing outcome data, selection of the reported result, and, in some studies, problems related to the randomization process. The outcome measurement domain was generally judged to be at low risk, reflecting the objective measurement of most outcomes. Figure 3 summarizes the distribution of the conservative study-level risk-of-bias judgments across the five RoB 2 domains. Detailed outcome-specific RoB 2 assessments are provided in Supplementary Table S3.

### 3.5. Adverse Events and Adherence

Of the 11 studies that reported adverse-event or safety outcomes, one study [29] reported transient nausea and vomiting in four participants immediately after training sessions during weeks 1–4. These events were mild to moderate, required no medical intervention, and resolved spontaneously. One study [42] reported two withdrawals: one due to an exercise-related injury and the other due to an illness unrelated to the intervention. Another study [44] reported five withdrawals, including two due to insufficient time, two for unknown reasons, and one due to an exercise-related injury. No serious adverse events were reported in the remaining studies [23,24,30,36,39,41,43,46].

Fifteen studies reported attendance or adherence data [23,24,29,30,32,33,34,38,39,41,43,44,45,47,50]. Reported rates ranged from 75.71% to 100%, and 12 studies reported rates of at least 90%. Based on the available point estimates and the lower bounds of threshold-only reports, the approximate unweighted mean was 92.7%. Reported attendance and adherence were generally high; however, the approximate mean should be interpreted descriptively because reporting formats differed across studies.

### 3.6. Meta-Analysis Results of HIIT Versus CON

**Figure 4:**
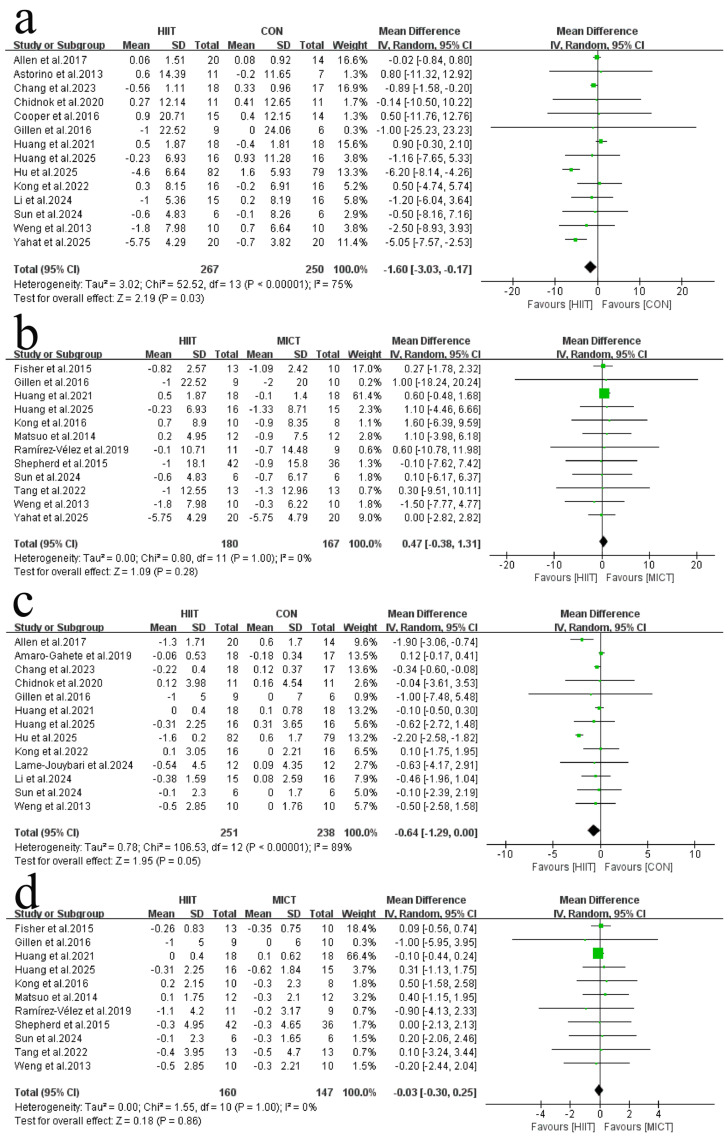
Forest plots of weighted mean differences for (**a**,**b**) body weight and (**c**,**d**) body mass index. (**a**,**c**) HIIT vs. CON; (**b**,**d**) HIIT vs. MICT. CON, non-exercise control; HIIT, high-intensity interval training; MICT, moderate-intensity continuous training. Green squares indicate individual study estimates, with their size reflecting study weights; black diamonds indicate pooled estimates with 95% confidence intervals.

**Figure 5:**
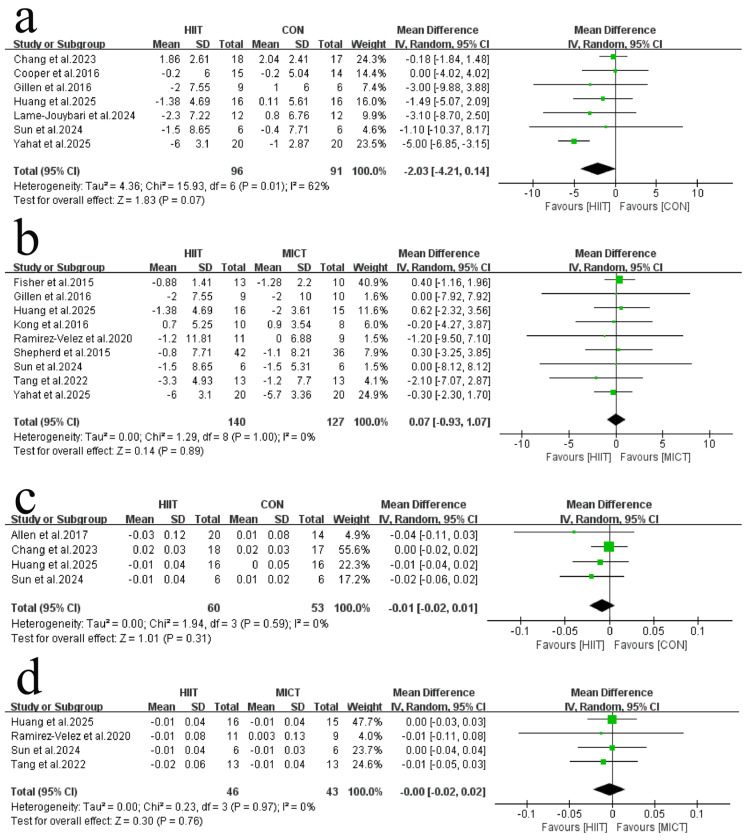
Forest plots of weighted mean differences for (**a**,**b**) body fat percentage and (**c**,**d**) waist-to-hip ratio. (**a**,**c**) HIIT vs. CON; (**b**,**d**) HIIT vs. MICT. CON, non-exercise control; HIIT, high-intensity interval training; MICT, moderate-intensity continuous training. Green squares indicate individual study estimates, with their size reflecting study weights; black diamonds indicate pooled estimates with 95% confidence intervals.

Supplementary Table S4 presents the pooled and subgroup analysis results for the comparison between HIIT and CON. Regarding body composition, HIIT significantly reduced BW (WMD = −1.60; 95% CI, −3.03 to −0.17; *p* = 0.03; Figure 4). The reduction in BMI did not reach statistical significance (WMD = −0.64, 95% CI, −1.29 to 0.00; *p* = 0.05; Figure 4), and no significant changes were observed in BF% or WHR (both *p* > 0.05; Figure 5). The certainty of evidence was rated as very low for all four outcomes (see Supplementary Table S5 for the reasons for downgrading).

Within-subgroup analyses identified significant reductions in BF% in the overweight/obesity subgroup, adolescent subgroup, extremely sedentary subgroup, and subgroups with a total session duration of ≤30 min or an intervention duration of <12 weeks (all *p* < 0.05). However, most subgroup estimates for BF% were based on fewer than three studies and should therefore be interpreted cautiously. For BW, significant reductions were observed in the adolescent, total session duration ≤30 min, and 2–3 sessions/week subgroups. No statistically significant reductions in BMI were observed in any subgroup, and no significant changes in WHR were observed in the total session duration subgroups.

**Figure 6:**
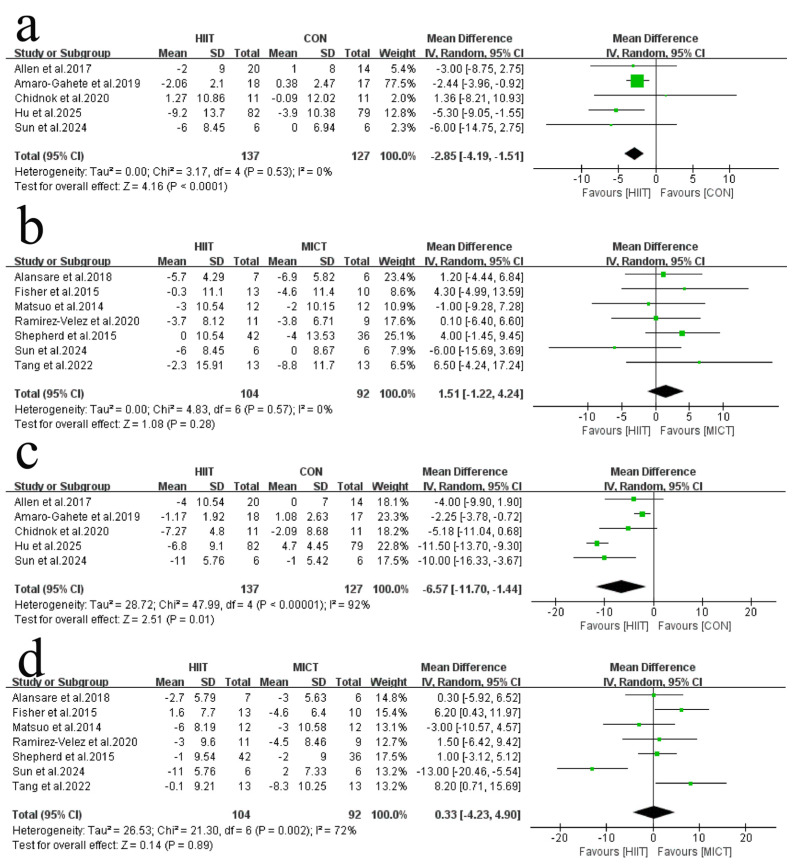
Forest plots of weighted mean differences for (**a**,**b**) systolic blood pressure and (**c**,**d**) diastolic blood pressure. (**a**,**c**) HIIT vs. CON; (**b**,**d**) HIIT vs. MICT. CON, non-exercise control; HIIT, high-intensity interval training; MICT, moderate-intensity continuous training. Green squares indicate individual study estimates, with their size reflecting study weights; black diamonds indicate pooled estimates with 95% confidence intervals.

**Figure 7:**
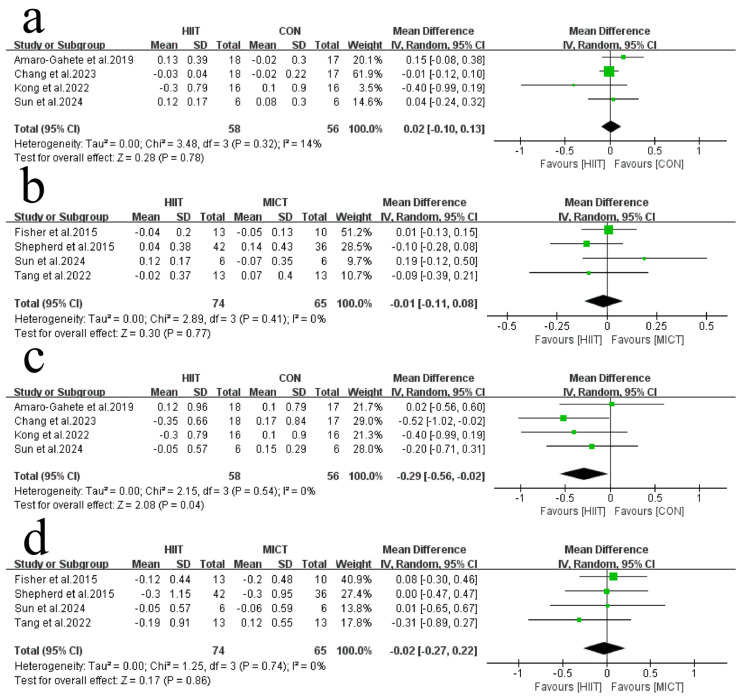
Forest plots of weighted mean differences for (**a**,**b**) high-density lipoprotein cholesterol and (**c**,**d**) low-density lipoprotein cholesterol. (**a**,**c**) HIIT vs. CON; (**b**,**d**) HIIT vs. MICT. CON, non-exercise control; HIIT, high-intensity interval training; MICT, moderate-intensity continuous training. Green squares indicate individual study estimates, with their size reflecting study weights; black diamonds indicate pooled estimates with 95% confidence intervals.

**Figure 8:**
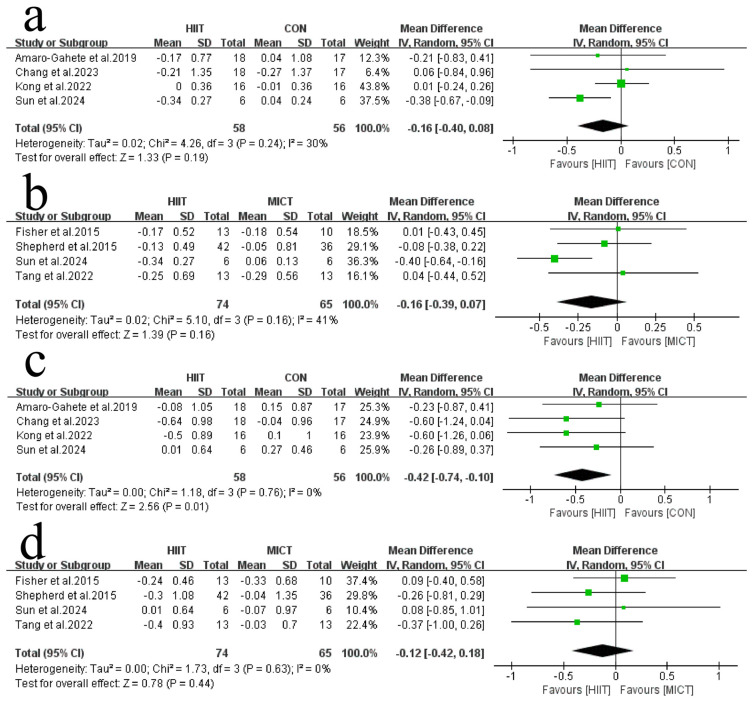
Forest plots of weighted mean differences for (**a**,**b**) triglycerides and (**c**,**d**) total cholesterol. (**a**,**c**) HIIT vs. CON; (**b**,**d**) HIIT vs. MICT. CON, non-exercise control; HIIT, high-intensity interval training; MICT, moderate-intensity continuous training. Green squares indicate individual study estimates, with their size reflecting study weights; black diamonds indicate pooled estimates with 95% confidence intervals.

**Figure 9:**
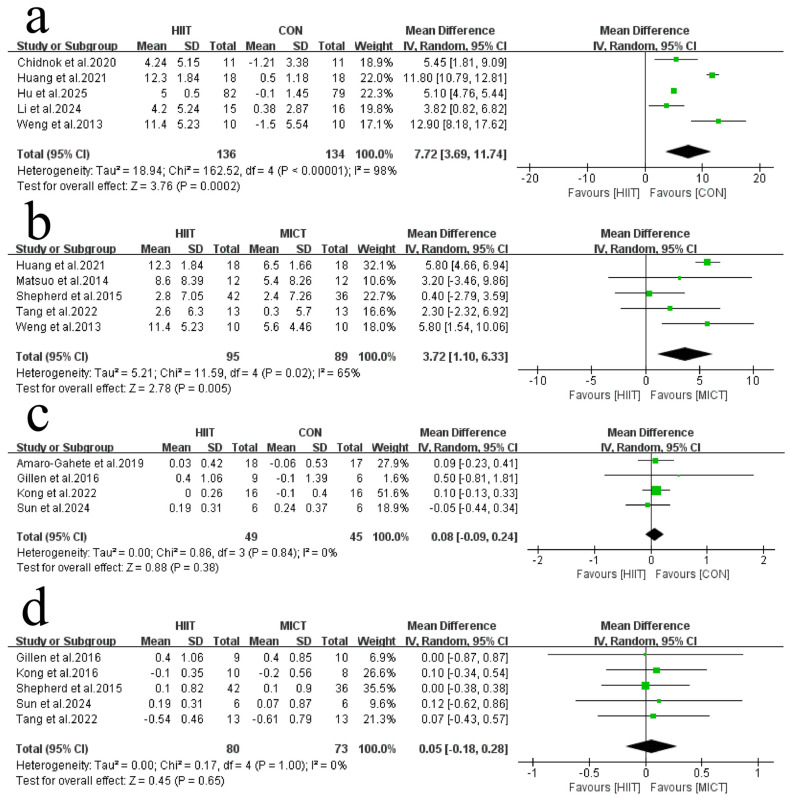
Forest plots of weighted mean differences for (**a**,**b**) maximal oxygen uptake and (**c**,**d**) blood glucose. (**a**,**c**) HIIT vs. CON; (**b**,**d**) HIIT vs. MICT. CON, non-exercise control; HIIT, high-intensity interval training; MICT, moderate-intensity continuous training. Green squares indicate individual study estimates, with their size reflecting study weights; black diamonds indicate pooled estimates with 95% confidence intervals.

Regarding cardiometabolic risk factors, HIIT significantly reduced SBP (WMD = −2.85 mmHg; 95% CI, −4.19 to −1.51; *p* < 0.0001; Figure 6), DBP (WMD = −6.57 mmHg; 95% CI, −11.70 to −1.44; *p* = 0.01; Figure 6), LDL-C (WMD = −0.29 mmol/L; 95% CI, −0.56 to −0.02; *p* = 0.04; Figure 7), and TC (WMD = −0.42 mmol/L; 95% CI, −0.74 to −0.10; *p* = 0.01; Figure 8) compared with CON. HIIT also significantly increased VO_2_max (WMD = 7.72 mL/kg/min; 95% CI, 3.69 to 11.74; *p* = 0.0002; Figure 9). No significant changes were observed in HDL-C, TG, or BG (all *p* > 0.05; Figure 7, Figure 8 and Figure 9). The certainty of evidence was rated as low for SBP and very low for all other outcomes (see Supplementary Table S5 for the reasons for downgrading).

Within-subgroup analyses showed significant reductions in SBP in subgroups defined by physical activity level, weight status, total session duration, and training frequency, as well as in the 12-week intervention subgroup and the middle-aged to older adults subgroup. However, no significant reductions in SBP were observed in the <12-week intervention, adolescent, or young adult subgroups. Significant reductions in DBP were observed in subgroups defined by physical activity level, weight status, age, total session duration, and training frequency, as well as in the 12-week intervention subgroup. Significant increases in VO_2_max were observed in all subgroups defined by total session duration, training frequency, and physical activity level (all *p* < 0.01). No significant changes in BG, TG, HDL-C, or LDL-C were observed in the total session duration or intervention duration subgroups. No significant changes in BG were observed in the weight status subgroups. Because most subgroup estimates were based on fewer than three studies, these findings should be interpreted cautiously.

### 3.7. Meta-Analysis Results of HIIT Versus MICT

Supplementary Table S6 presents the pooled and subgroup analysis results for the comparison between HIIT and MICT. No significant between-group differences were observed for body composition or most cardiometabolic outcomes. However, HIIT produced a significantly greater increase in VO_2_max than MICT (WMD = 3.72 mL/kg/min; 95% CI, 1.10 to 6.33; *p* = 0.005; Figure 9). The certainty of evidence was rated as low for BF%, BG, and VO_2_max and very low for all other outcomes (see Supplementary Table S5 for the reasons for downgrading).

Exploratory within-subgroup analyses showed no clear differences in VO_2_max between HIIT and MICT in the total session duration ≤30 min, 2–3 sessions/week, or insufficient physical activity alone subgroups. In contrast, within-subgroup estimates significantly favored HIIT in the total session duration >30 min, 4–5 sessions/week, and extremely sedentary subgroups (*p* < 0.00001). However, the tests for subgroup differences were not statistically significant; therefore, these findings should not be interpreted as evidence of effect modification. A significantly greater reduction in TG with HIIT was also observed in the total session duration ≤30 min subgroup (*p* = 0.008), although this estimate was based on only three studies. Other subgroup comparisons showed no clear between-group differences. Because several subgroup estimates were based on fewer than three studies, all subgroup findings should be considered exploratory and interpreted cautiously.

### 3.8. Sensitivity Analyses and Publication Bias

Leave-one-out sensitivity analyses showed that the pooled results were robust for most outcomes, whereas the results for BW, LDL-C, TC, BMI, BF%, VO_2_max, and TG were sensitive to the removal of individual studies. For the comparison with CON, the significant reduction in BW was no longer observed after the individual removal of Chang et al. (2023) [23], Hu et al. (2025) [29], or Yahat (2025) [49]. Similarly, the significant reductions in LDL-C and TC were no longer observed after the removal of specific studies [23,35]. Conversely, the pooled reduction in BMI became statistically significant after the removal of specific studies [35,44]. The pooled reductions in BF% and TG also became statistically significant after the removal of Chang et al. (2023) [23] and Kong et al. (2022) [35], respectively. For the comparison with MICT, the significant advantage of HIIT for VO_2_max was no longer observed after the removal of Weng et al. (2013) [39]. Visual inspection of the funnel plots did not indicate clear asymmetry (see Supplementary File S2).

## 4. Discussion

### 4.1. Principal Findings

This systematic review and meta-analysis focused on physically inactive individuals and included an exploratory analysis of evidence from extremely sedentary populations. Compared with CON, HIIT was associated with a modest reduction in BW, reductions in SBP, DBP, TC, and LDL-C, and an increase in VO_2_max. Exploratory within-subgroup analyses suggested reductions in BF% in the overweight/obesity, adolescent, and extremely sedentary subgroups, as well as in subgroups with a total session duration of ≤30 min or an intervention duration of <12 weeks. Most of these BF% findings were based on fewer than three studies and should therefore be interpreted cautiously. Reductions in BW were also observed in the adolescent, total session duration ≤30 min, and 2–3 sessions/week subgroups, although the pooled BW result was sensitive to the removal of individual studies. Compared with MICT, no statistically significant between-group differences were observed for most outcomes, whereas HIIT was associated with a greater increase in VO_2_max. Across both comparator analyses, improvement in VO_2_max emerged as a relatively consistent finding. However, the certainty of evidence for VO_2_max was very low for the comparison with CON and low for the comparison with MICT, and the HIIT-versus-MICT result was sensitive to the omission of a single study. Exploratory within-subgroup analyses favored HIIT for VO_2_max in the total session duration >30 min and 4–5 sessions/week subgroups, but not in the total session duration ≤30 min or 2–3 sessions/week subgroups. However, the tests for subgroup differences were not statistically significant, so these patterns should not be interpreted as evidence of effect modification. Among the 11 studies reporting safety outcomes, no serious adverse events were reported, and reported attendance or adherence was generally high, although these findings were limited by incomplete and heterogeneous reporting and the predominance of supervised interventions.

### 4.2. Effects of HIIT on Body Composition

In this meta-analysis, HIIT was associated with a modest reduction in BW. However, this finding was sensitive to the removal of individual studies, and the certainty of evidence was very low. It should therefore be interpreted with caution. No statistically significant overall changes were observed in BMI, WHR, or BF%. Compared with MICT, HIIT showed no statistically significant between-group differences in body composition outcomes; however, the absence of significant differences should not be interpreted as evidence of equivalence. One possible explanation is that BW reflects total body mass and does not distinguish among fat mass, lean tissue, and body fluids. A modest change in BW may therefore occur without corresponding changes in fat mass or fat distribution [51,52]. In addition, the included studies mainly reported BF% or fat mass, with lean body mass reported infrequently, and used different methods to assess body composition. Differences in the reported components and assessment methods may have contributed to variation across studies [53,54]. Consequently, it remains unclear which body component accounted for the observed reduction in BW. Similarly, the absence of a statistically significant change in BMI cannot be used to infer a specific energy-related mechanism or the preservation of lean body mass. Larger randomized controlled trials using comprehensive and standardized body composition assessments are needed to examine these possibilities.

Exploratory within-subgroup analyses showed significant reductions in BF% in the overweight/obesity, adolescent, extremely sedentary, total session duration ≤30 min, and intervention duration <12 weeks subgroups. Significant reductions in BW were observed in the adolescent, total session duration ≤30 min, and 2–3 sessions/week subgroups. Greater baseline adiposity or less previous exercise exposure may increase responsiveness to a novel exercise stimulus and make early adaptations more detectable. This interpretation is consistent with the proposed concepts of a “novel stimulus” and “diminishing returns” [55]. However, the formal test for subgroup differences did not establish physical activity status as an effect modifier, so this explanation remains speculative. Two randomized controlled trials also reported reductions in fat mass, BF%, or visceral fat after HIIT in sedentary adolescents and college students with obesity. These findings are directionally consistent with some of the subgroup results in the present review [30,56]. However, most BF% subgroup estimates were based on fewer than three studies. With the exception of the BW analysis by total session duration, tests for subgroup differences were not statistically significant. These subgroup findings therefore do not establish that HIIT is more effective in any specific population or intervention setting.

For BW, the test for subgroup differences by total session duration was statistically significant. The reduction was greater in the ≤30 min subgroup than in the >30 min subgroup (Chi^2^ = 7.88, df = 1, *p* = 0.005, I^2^ = 87.3%). This finding raises the possibility that the effect of HIIT on BW may vary according to total session duration. However, the available data do not explain the basis of this difference. In this review, total session duration included the warm-up, interval-training and recovery periods, and cool-down. It therefore reflected the time required to complete a full HIIT session rather than the actual time spent at high intensity. Total session duration should therefore not be considered a precise measure of HIIT dose. Accordingly, the current evidence does not establish that shorter sessions are superior to longer sessions. Future studies should report total session duration, accumulated high-intensity work time, recovery time, and total training load.

### 4.3. Effects of HIIT on Blood Pressure

Previous evidence suggests that physical inactivity and sedentary behavior may be associated with an increased risk of hypertension [57]. In this meta-analysis, HIIT was associated with reductions in SBP and DBP compared with CON, consistent with a previous meta-analysis [58]. However, the certainty of evidence was low for SBP and very low for DBP. In addition, most participants did not have a diagnosis of hypertension. These findings should therefore not be interpreted as direct evidence that HIIT is an effective treatment for hypertension. Exploratory within-subgroup analyses showed significant reductions in SBP in all subgroups defined by physical activity level, weight status, total session duration, and training frequency. Significant reductions were also observed in the 12-week intervention subgroup and the middle-aged to older adults subgroup. Significant reductions in DBP were observed in all subgroups defined by physical activity level, weight status, age, total session duration, and training frequency, as well as in the 12-week intervention subgroup. Despite these within-subgroup findings, the tests for subgroup differences were not statistically significant. These results therefore do not establish that the effects differed across populations or intervention protocols. Previous studies suggest that the hypotensive effect of HIIT may involve improved endothelial function and autonomic regulation, reduced peripheral vascular resistance, and increased arterial compliance [58,59,60]. These adaptations may jointly improve vasodilation and hemodynamic regulation. However, most included studies did not directly assess these mechanisms. The current evidence therefore supports a possible hypotensive effect of HIIT but does not identify the optimal total session duration, training frequency, or intervention duration. Future studies should examine whether the hypotensive effect of HIIT follows a linear or nonlinear dose–response pattern. They should also consider accumulated high-intensity work time, recovery time, and total training load when evaluating the safety and effectiveness of HIIT protocols, particularly in individuals with hypertension who also exhibit sedentary behavior.

### 4.4. Effects of HIIT on Cardiorespiratory Fitness

Previous research suggests that physical inactivity is generally associated with lower cardiorespiratory fitness [61]. In this meta-analysis, HIIT was associated with a significant increase in VO_2_max compared with CON. HIIT also produced a significantly greater increase in VO_2_max than MICT, consistent with the overall conclusion of a previous umbrella review [12]. However, the certainty of evidence for VO_2_max was very low for the comparison with CON. For the comparison with MICT, the statistically significant between-group difference was no longer observed after Weng et al. (2013) [39] was removed in the leave-one-out sensitivity analysis, and the certainty of evidence was low. Therefore, whether HIIT provides a consistent advantage over MICT for improving VO_2_max remains uncertain. Exploratory within-subgroup analyses favored HIIT over MICT for VO_2_max in the total session duration >30 min, 4–5 sessions/week, and extremely sedentary subgroups. A lower baseline level of cardiorespiratory fitness in extremely sedentary individuals could theoretically allow greater relative improvement. A longer total session duration and higher training frequency may also increase overall training exposure or the cumulative physiological stimulus [14,62]. However, the tests for subgroup differences were not statistically significant. These subgroup findings therefore do not establish a dose–response relationship or identify an optimal HIIT protocol.

Across the included comparisons, HIIT generally involved shorter training sessions than MICT, suggesting a potential time-efficiency advantage. However, training volume and energy expenditure were not consistently matched across studies. In addition, the advantage of HIIT over MICT for VO_2_max was sensitive to the removal of one study. It therefore remains unclear whether HIIT produces greater improvements in cardiorespiratory fitness than MICT while requiring less training time. The increase in VO_2_max after HIIT may involve both central and peripheral physiological adaptations. Previous research suggests that HIIT may increase maximal stroke volume and cardiac output. HIIT may also increase skeletal muscle mitochondrial content and oxidative enzyme activity, although findings for other peripheral adaptations have been less consistent [14,63]. The physiological stimulus elicited by HIIT may vary according to the intensity and duration of the work intervals. During short all-out exercise bouts, the phosphagen and glycolytic systems generally make greater relative contributions to energy provision. As the duration of an exercise bout increases, the relative contribution of oxidative metabolism generally increases. Nevertheless, all three energy systems contribute throughout maximal exercise [64]. Thus, although HIIT protocols differ in their energy-system demands, each may still provide a stimulus for aerobic adaptation. This may partly explain why the pooled analysis showed an increase in VO_2_max despite substantial variation across HIIT protocols. However, the included studies did not consistently assess these physiological mechanisms, so this explanation remains speculative. Future studies should distinguish between different types of HIIT and report accumulated high-intensity work time, recovery time, total session duration, accumulated time spent at or near VO_2_max, and total training load.

### 4.5. Effects of HIIT on Lipid and Glucose Metabolism

In this meta-analysis, HIIT was associated with significant reductions in TC and LDL-C compared with CON, whereas no significant changes were observed in BG, TG, or HDL-C. Compared with MICT, no statistically significant between-group differences were observed for any lipid or glucose outcome. The significant findings for TC and LDL-C were sensitive to the removal of individual studies, and the certainty of evidence for the lipid and glucose outcomes was low or very low. Therefore, the effects of HIIT on lipid and glucose metabolism remain uncertain. Exploratory subgroup analyses showed no consistent pattern across populations or intervention characteristics, and most subgroup estimates were based on few studies. Several physiological mechanisms may contribute to the metabolic responses to HIIT. Muscle glycogen depletion and subsequent glycogen resynthesis, together with AMP-activated protein kinase (AMPK) activation and glucose transporter type 4 (GLUT4) translocation, may increase skeletal muscle glucose uptake after HIIT. Catecholamine responses may increase fatty acid mobilization, whereas mitochondrial adaptations may enhance fatty acid oxidation capacity [65,66]. However, acute high-intensity exercise generally relies heavily on carbohydrate metabolism, and acute changes in substrate use or longer-term tissue adaptations may not translate directly into measurable changes in fasting BG or circulating lipid levels [66]. In addition, the included interventions were limited to 2–12 weeks, and dietary control, blood-sampling timing, and fasting conditions varied across studies. These factors may have contributed to between-study variability and reduced the ability to detect sustained metabolic changes [67,68,69]. In addition, most participants did not have a diagnosed metabolic disease, and their baseline BG and lipid levels may have been within or near normal ranges, which may have limited the magnitude of detectable change. Previous studies suggest that higher baseline fasting glucose, LDL-C, and TG levels may be associated with larger reductions after exercise training [70,71,72]. However, the included studies did not directly or consistently assess these mechanisms, and baseline metabolic status was not formally tested as an effect modifier. These explanations therefore remain speculative. Future studies should use longer intervention and follow-up periods, standardize dietary control and blood-sampling conditions, and assess the effects of HIIT on lipid and glucose metabolism using designs that can isolate the independent contribution of HIIT.

### 4.6. Safety and Adherence

Among the 11 studies that reported safety outcomes, the reported events consisted mainly of transient mild-to-moderate nausea or vomiting and a small number of exercise-related injuries; no serious adverse events were reported. The available data suggest that HIIT was generally well tolerated in the studies that reported safety outcomes. However, safety reporting was incomplete, and most interventions were short-term and supervised. The safety of unsupervised, higher-frequency, or longer-term HIIT therefore remains uncertain. Fifteen studies reported attendance or adherence rates ranging from 75.71% to 100%. Twelve studies reported rates of at least 90%, and the approximate unweighted mean was 92.7%. Reported attendance and adherence were therefore generally high. However, reporting definitions and formats varied across studies, so the mean should be interpreted descriptively. The high reported rates may also have been influenced by close supervision and the short intervention periods. A previous systematic review similarly found high completion rates in supervised HIIT studies, whereas long-term adherence in unsupervised settings was lower and more heterogeneous [73].

Because the included evidence was not derived from disease-specific cardiac rehabilitation populations, these findings cannot be directly extrapolated to cardiac rehabilitation settings. The findings may nevertheless inform supervised and individualized exercise prescription for physically inactive individuals, but they do not establish the safety or effectiveness of HIIT in disease-specific rehabilitation populations. The predominance of short-term, supervised interventions also limits generalizability to long-term and real-world settings. Accordingly, a cautious and individualized progression of HIIT intensity and frequency may be appropriate for physically inactive individuals, taking account of health status, previous exercise experience, and pre-exercise risk screening. Appropriate warm-up, cool-down, and supervision should also be considered, particularly during initial participation. Current evidence remains insufficient to recommend any specific total session duration, training frequency, or HIIT type as universally optimal.

### 4.7. Strengths and Limitations of the Study

This review has several strengths. It focused specifically on physically inactive individuals and included exploratory analyses of populations classified as extremely sedentary. A total of 24 randomized controlled trials were included, covering body composition, blood pressure, lipid and glucose metabolism, cardiorespiratory fitness, safety, and adherence. HIIT was evaluated separately against CON and MICT, and random-effects models were used for all meta-analyses. Risk of bias, certainty of evidence, and result robustness were assessed using RoB 2, GRADE, and leave-one-out sensitivity analyses, respectively. In addition, the use of WMDs allowed intervention effects to be expressed on their original measurement scales, facilitating clinical interpretation.

Several limitations should be considered when interpreting the findings. Conference abstracts and dissertations were excluded, and only one trial registry was searched; although no eligible unpublished studies were identified in the sources searched, publication bias and other dissemination-related biases cannot be ruled out because other grey literature sources were not systematically examined. The certainty of evidence was low or very low for most outcomes, and several pooled results were sensitive to the removal of individual studies. Furthermore, blinding of participants and intervention providers was generally not feasible in exercise interventions, which may have introduced performance bias, particularly for outcomes influenced by participant behavior or motivation. The broad age range of the included participants, encompassing age groups with distinct physiological characteristics, and the substantial variation in HIIT protocols may have introduced additional heterogeneity. Because the number of studies and participants was limited for many outcomes and comparisons, several subgroup estimates had limited precision and statistical power, and reliable subgroup analyses according to HIIT type could not be performed. Although evidence cited in the Introduction suggests that physical inactivity may be more prevalent among women than men, few studies reported sex-stratified outcomes, preventing the evaluation of potential sex-related differences in responses to HIIT. The session-duration subgroups were based on total session duration rather than accumulated high-intensity work time and therefore should not be interpreted as a precise measure of HIIT dose. In addition, the classification of extremely sedentary populations relied mainly on descriptions of sedentary behavior, questionnaire-based assessments, or reported sedentary time, and only two studies provided a specific sedentary-time threshold. The absence of a common objective threshold may have introduced classification error. This subgroup analysis was therefore exploratory, and its findings should be interpreted cautiously. Finally, the interventions lasted 2–12 weeks, and long-term follow-up data were limited, restricting the assessment of long-term effects, safety, and sustained adherence.

Future studies should use standardized and clearly differentiated definitions of physical inactivity and sedentary behavior and, where feasible, measure sedentary time objectively. HIIT protocols should be reported in sufficient detail, including HIIT type, accumulated high-intensity work time, recovery time, total session duration, training frequency, and total training load. Key outcomes and planned subgroup analyses should be prespecified, and adequately powered randomized controlled trials with longer follow-up and sex-stratified reporting are needed to determine whether effects differ across populations and protocols. Research conducted in real-world settings should also compare supervised and unsupervised HIIT and standardize the definitions, monitoring, and reporting of adverse events, attendance, and adherence.

## 5. Conclusions

This meta-analysis suggests that HIIT may improve selected body composition and cardiometabolic outcomes in physically inactive individuals. Compared with CON, HIIT was associated with modest reductions in BW, SBP, DBP, TC, and LDL-C, as well as an increase in VO_2_max. Compared with MICT, no statistically significant between-group differences were observed for most outcomes, whereas HIIT was associated with a greater increase in VO_2_max. Across the two comparator analyses, improvement in VO_2_max was a relatively consistent finding. These findings support further investigation of HIIT as a potentially time-efficient exercise strategy for health promotion among physically inactive individuals. However, the certainty of evidence was low or very low for most outcomes, and the findings for BW, TC, and LDL-C versus CON, as well as VO_2_max versus MICT, were sensitive to the removal of individual studies.

Reported safety and adherence outcomes were generally favorable; however, safety reporting was incomplete, and most interventions were short-term and supervised. Current evidence does not establish equivalence between HIIT and MICT and is insufficient to identify an optimal HIIT protocol. Larger randomized controlled trials with longer intervention and follow-up periods are needed and should also standardize the reporting of HIIT dose, objectively measure sedentary time, provide sex-stratified outcomes, and systematically monitor adverse events, attendance, and adherence. Findings from extremely sedentary populations should be considered exploratory until more directly targeted trials become available.

## Data Availability

The data supporting the findings of this systematic review and meta-analysis are included in the article and Supplementary Materials. The raw data for funnel plot generation, subgroup analyses of body composition outcomes, subgroup analyses of cardiometabolic risk factor outcomes, and all outcomes are provided in Supplementary Files S3–S6. Further inquiries can be directed to the corresponding author.

## Supplementary Materials

The following supporting information can be downloaded at https://www.mdpi.com/article/10.3390/metabo16070514/s1: Supplementary File S1: Search Strategies for All Databases; Supplementary Table S1: Summary of included study characteristics; Supplementary File S2: Funnel plots for publication bias assessment. Funnel plots are shown only for outcomes with at least 10 studies included in the meta-analysis. The four plots present body mass index and body weight for HIIT versus CON and HIIT versus MICT comparisons. WMD, weighted mean difference; HIIT, high-intensity interval training; CON, control; MICT, moderate-intensity continuous training; Supplementary Table S2: Summary of HIIT and MICT design protocols across studies; Supplementary File S3: Raw data for generating funnel plots; Supplementary Table S3: Outcome-Specific RoB 2 Assessments of Included Studies; Supplementary File S4: Raw data for subgroup analysis of body composition indicators; Supplementary Table S4: Summary of meta-analyses on high-intensity interval training versus no-exercise control group; Supplementary File S5: Raw data for subgroup analysis of cardiometabolic risk factor indicators; Supplementary Table S5: Summary of results and evaluation of the certainty of evidence (GRADE); Supplementary File S6: Raw data for all indicators; Supplementary Table S6: Summary of meta-analyses on high-intensity interval training versus moderate-intensity continuous training.

## Abbreviations

HIIT	high-intensity interval training
MICT	moderate-intensity continuous training
CON	non-exercise control
WMD	weighted mean difference
BW	body weight
BF%	body fat percentage
BMI	body mass index
WHR	waist-to-hip ratio
BG	blood glucose
TG	triglycerides
TC	total cholesterol
SBP	systolic blood pressure
DBP	diastolic blood pressure
HDL-C	high-density lipoprotein cholesterol
LDL-C	low-density lipoprotein cholesterol
VO_2_max	maximal oxygen uptake
EPOC	excess post-exercise oxygen consumption
GRADE	Grading of Recommendations Assessment, Development and Evaluation
AMPK	AMP-activated protein kinase
GLUT4	glucose transporter type 4
IPAQ	International Physical Activity Questionnaire
HRmax	maximum heart rate
