# Supplementary material for: Effects of High-Intensity Interval Training on Body Composition and Cardiometabolic Health in Physically Inactive Individuals: A Systematic Review and Meta-Analysis of Randomized Controlled Trials
Source: Metabolites. 2026 Jul 22;16(7):514. doi: 10.3390/metabo16070514 (PMC13413877; doi:10.3390/metabo16070514)
Supplement: Supplementary file 1 [file metabolites-16-00514-s001.zip › Supplementary File S1.pdf]

Supplementary File S1. Search Strategies for All Databases

PubMed:

#1： (((((((((((High-Intensity Interval Training [MeSH Terms]) OR (High Intensity Interval Training[Title/Abstract])) OR (High-Intensity Interval Trainings[Title/Abstract])) OR(Interval Training, High-Intensity[Title/Abstract])) OR (Interval Trainings, High-Intensity[Title/Abstract])) OR (Training, High-Intensity Interval[Title/Abstract])) OR (Trainings, High-Intensity Interval[Title/Abstract])) OR (High-Intensity Intermittent Exercise[Title/Abstract])) OR (Exercise, High-Intensity Intermittent[Title/Abstract])) OR (Exercises, High-Intensity Intermittent[Title/Abstract])) OR (High-Intensity Intermittent Exercises[Title/Abstract])) OR (Sprint Interval Training[Title/Abstract])) OR (Sprint Interval Trainings[Title/Abstract])

#2 ： (((((((((Sedentary Behavior[MeSH Terms]) OR (Behavior, Sedentary[Title/Abstract])) OR (Sedentary Behaviors[Title/Abstract])) OR (Sedentary Lifestyle[Title/Abstract])) OR (Lifestyle, Sedentary[Title/Abstract])) OR (Physical Inactivity[Title/Abstract])) OR (Inactivity, Physical[Title/Abstract])) OR (Lack of Physical Activity[Title/Abstract])) OR (Sedentary Time[Title/Abstract])) OR (Sedentary Times[Title/Abstract])) OR (Time, Sedentary[Title/Abstract])

#3: #1 AND #2

(((((((((((High-Intensity Interval Training[MeSH Terms]) OR (High Intensity Interval Training[Title/Abstract])) OR (High-Intensity Interval Trainings[Title/Abstract])) OR (Interval Training, High-Intensity[Title/Abstract])) OR (Interval Trainings, High-

Intensity[Title/Abstract])) OR (Training, High-Intensity Interval[Title/Abstract])) OR (Trainings, High-Intensity Interval[Title/Abstract])) OR (High-Intensity Intermittent Exercise[Title/Abstract])) OR (Exercise, High-Intensity Intermittent[Title/Abstract])) OR (Exercises, High-Intensity Intermittent[Title/Abstract])) OR (High-Intensity Intermittent Exercises[Title/Abstract])) OR (Sprint Interval Training[Title/Abstract])) OR (Sprint Interval Trainings[Title/Abstract]) AND (((((((((Sedentary Behavior[MeSH Terms]) OR (Behavior, Sedentary[Title/Abstract])) OR (Sedentary Behaviors[Title/Abstract])) OR (Sedentary Lifestyle[Title/Abstract])) OR (Lifestyle, Sedentary[Title/Abstract])) OR (Physical Inactivity[Title/Abstract])) OR (Inactivity, Physical[Title/Abstract])) OR (Lack of Physical Activity[Title/Abstract])) OR (Sedentary Time[Title/Abstract])) OR (Sedentary Times[Title/Abstract])) OR (Time, Sedentary[Title/Abstract])

Web of Science:

#1: ((((((((((((ALL=(High-Intensity Interval Training)) OR TS=(High Intensity Interval Training)) OR TS=(High-Intensity Interval Trainings)) OR TS=(Interval Training, High-Intensity)) OR TS=(Interval Trainings, High-Intensity)) OR TS=(Training, High-Intensity Interval)) OR TS=(Trainings, High-Intensity Interval)) OR TS=(High-Intensity Intermittent Exercise)) OR TS=(Exercise, High-Intensity Intermittent)) OR TS=(Exercises, High-Intensity Intermittent)) OR TS=(High-Intensity Intermittent Exercises)) OR TS=(Sprint Interval Training)) OR TS=(Sprint Interval Trainings)

#2: ((((((((((ALL=(Sedentary Behavior)) OR TS=(Behavior, Sedentary)) OR TS=(Sedentary

Behaviors)) OR TS=(Sedentary Lifestyle)) OR TS=(Lifestyle, Sedentary)) OR TS=(Physical Inactivity)) OR TS=(Inactivity, Physical)) OR TS=(Lack of Physical Activity)) OR TS=(Sedentary Time)) OR TS=(Sedentary Times)) OR TS=(Time, Sedentary)

#3: #1 AND #2

Embase:

#1: 'high intensity interval training' OR 'high-intensity intermittent exercise' OR 'high-intensity intermittent training' OR 'high-intensity interval exercise' OR 'high-intensity interval training' OR 'HIIE (exercise)' OR 'HIIT' OR 'intermittent high-intensity training' OR 'interval high-intensity training' OR 'high intensity interval training'

#2: 'sedentary lifestyle' OR 'sedentary behavior' OR 'sedentary behaviour' OR 'sedentary life style' OR 'sedentary lifestyle' OR 'physical inactivity' OR 'physical inactivity'

#3: #1 AND #2

Cochrane Library:

#1: High-Intensity Interval Training OR Exercise, High-Intensity Intermittent OR High-Intensity Interval Trainings OR High-Intensity Intermittent Exercises OR Training, High-Intensity Interval OR Trainings, High-Intensity Interval OR Interval Training, High-Intensity OR High Intensity Interval Training OR Interval Trainings, High-Intensity OR High-Intensity Intermittent Exercise OR Exercises, High-Intensity Intermittent OR Sprint Interval Trainings OR Sprint Interval Training

#2: Sedentary Behavior OR Synonyms Lack of Physical Activity OR Inactivity, Physical OR

Physical Inactivity OR Sedentary Behaviors OR Lifestyle, Sedentary OR Behavior, Sedentary OR Sedentary Lifestyle OR Sedentary Times OR Sedentary Time OR Time, Sedentary

#3: #1 AND #2

Ovid Resources Searched [Ovid MEDLINE; APA PsycINFO (all available coverage files); APA PsycArticles Full Text Database; Books@Ovid; and Subscribed Journals Full Text.]:

#1: (High-Intensity Interval Training or (high-intensity interval training or high intensity interval training or high-intensity interval trainings or interval training, high-intensity or interval trainings, high-intensity or training, high-intensity interval or trainings, high-intensity interval or high-intensity intermittent exercise or exercise, high-intensity intermittent or exercises, high-intensity intermittent or high-intensity intermittent exercises or sprint interval training or sprint interval trainings)).mp.

#2: (Sedentary Behavior or (sedentary behavior or behavior, sedentary or sedentary behaviors or sedentary lifestyle or lifestyle, sedentary or physical inactivity or inactivity, physical or "lack of physical activity" or sedentary time or sedentary times or time, sedentary)).mp.

#3: #1AND #2

ClinicalTrials.gov:

(AREA[InterventionName]"high intensity interval training" OR AREA[InterventionName]"high-intensity interval training" OR AREA[InterventionName]HIIT OR AREA[InterventionName]"sprint interval training" OR AREA[InterventionName]"sprint interval exercise" OR AREA[InterventionName]"high intensity intermittent exercise" OR

AREA[InterventionName]"high-intensity intermittent exercise" OR AREA[BriefSummary]"high intensity interval training" OR AREA[BriefSummary]"high-intensity interval training" OR AREA[BriefSummary]"sprint interval training" OR AREA[DetailedDescription]"high intensity interval training" OR AREA[DetailedDescription]"high-intensity interval training" OR AREA[DetailedDescription]"sprint interval training")

AND

(AREA[BasicSearch]sedentary OR AREA[BasicSearch]"sedentary behavior" OR AREA[BasicSearch]"sedentary lifestyle" OR AREA[BasicSearch]"physical inactivity" OR AREA[BasicSearch]"physically inactive" OR AREA[BasicSearch]"insufficiently active" OR AREA[BasicSearch]"low physical activity" OR AREA[BasicSearch]inactive OR AREA[BasicSearch]untrained)
