# Supplementary material for: Effects of High-Intensity Interval Training on Body Composition and Cardiometabolic Health in Physically Inactive Individuals: A Systematic Review and Meta-Analysis of Randomized Controlled Trials
Source: Metabolites. 2026 Jul 22;16(7):514. doi: 10.3390/metabo16070514 (PMC13413877; doi:10.3390/metabo16070514)

Supplementary File S2. Funnel plots for publication bias assessment. Funnel plots are shown only for outcomes with at least 10 studies included in the meta-analysis. The four plots present body mass index and body weight for HIIT versus CON and HIIT versus MICT comparisons. WMD, weighted mean difference; HIIT, high-intensity interval training; CON, control; MICT, moderate-intensity continuous training.

HIIT vs CON

1. Body mass index

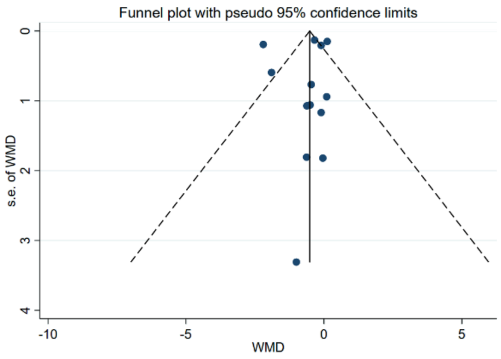

2. Body weight

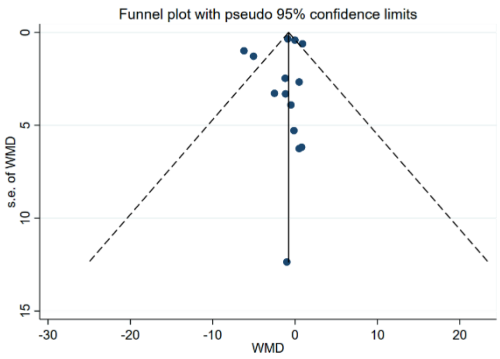

HIIT vs MICT

1. Body mass index

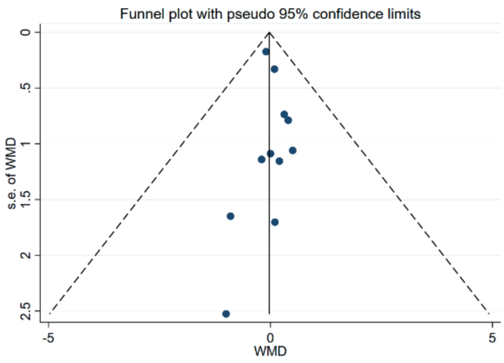

2. Body weight

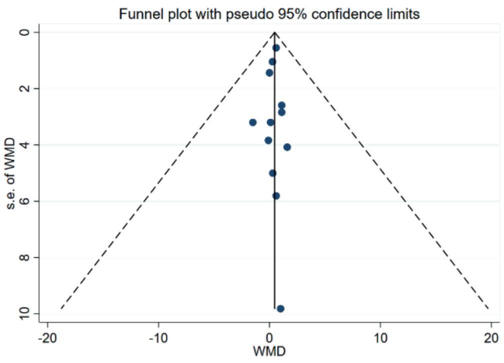

Supplement: Supplementary file 1 [file metabolites-16-00514-s001.zip › Supplementary File S2.pdf]
